# Intracranial Fluid Redistribution But No White Matter Microstructural Changes During a Spaceflight Analog

**DOI:** 10.1038/s41598-017-03311-w

**Published:** 2017-06-09

**Authors:** Vincent Koppelmans, Ofer Pasternak, Jacob J. Bloomberg, Yiri E. De Dios, Scott J. Wood, Roy Riascos, Patricia A. Reuter-Lorenz, Igor S. Kofman, Ajitkumar P. Mulavara, Rachael D. Seidler

**Affiliations:** 1 School of Kinesiology, University of Michigan, Ann Arbor, MI United States; 2 Departments of Psychiatry and Radiology, Brigham and Women’s Hospital and Harvard Medical School, Boston, MA United States; 3 NASA Johnson Space Center, Houston, TX United States; 4 KBRwyle, Houston, TX United States; 5 Azusa Pacific University, Azusa, CA United States; 6 The University of Texas Health Science Center, Houston, TX United States; 7 Department of Psychology, University of Michigan, Ann Arbor, MI United States; 8 Neuroscience Program, University of Michigan, Ann Arbor, MI United States

## Abstract

The neural correlates of spaceflight-induced sensorimotor impairments are unknown. Head down-tilt bed rest (HDBR) serves as a microgravity analog because it mimics the headward fluid shift and axial body unloading of spaceflight. We investigated focal brain white matter (WM) changes and fluid shifts during 70 days of 6° HDBR in 16 subjects who were assessed pre (2x), during (3x), and post-HDBR (2x). Changes over time were compared to those in control subjects (n = 12) assessed four times over 90 days. Diffusion MRI was used to assess WM microstructure and fluid shifts. Free-Water Imaging was used to quantify distribution of intracranial extracellular free water (FW). Additionally, we tested whether WM and FW changes correlated with changes in functional mobility and balance measures. HDBR resulted in FW increases in fronto-temporal regions and decreases in posterior-parietal regions that largely recovered by two weeks post-HDBR. WM microstructure was unaffected by HDBR. FW decreases in the post-central gyrus and precuneus correlated negatively with balance changes. We previously reported that gray matter increases in these regions were associated with less HDBR-induced balance impairment, suggesting adaptive structural neuroplasticity. Future studies are warranted to determine causality and underlying mechanisms.

## Introduction

Long-duration spaceflight can result in changes in gait and mobility that have been associated with different spaceflight-induced physiological changes^1–3^. Recent longitudinal neuroimaging studies in astronauts have shown that brain structure and functional connectivity are also affected by spaceflight^4, 5^. For example, we recently reported that astronauts exhibit volumetric gray matter decreases, as measured by anatomical MRI, including large areas covering the temporal and frontal poles and around the orbits, from pre-to-post spaceflight^5^. Furthermore, astronauts demonstrated bilateral focal gray matter increases in cerebral areas where the lower limbs are represented (i.e., the medial primary somatosensory and motor cortex). This is interesting considering the sensorimotor adaptation that occurs with spaceflight and associated static and dynamic balance problems that astronauts experience post flight. However, we did not observe correlations between brain changes and changes in balance performance in that retrospective sample^5^.

These effects of spaceflight on brain structure and function are in line with findings reported from long-duration head down tilt bed rest (HDBR) neuroimaging studies. HDBR studies serve as a microgravity analog because they mimic the headward fluid shifts and body unloading of spaceflight^6^. Recent HDBR studies from our lab as well as others utilizing anatomical functional and diffusion MRI showed that HDBR leads to gray matter increases in posterior parietal areas and decreases in fronto-temporal brain areas^5, 7, 8^, changes in white matter microstructure^7^, changes in the functional connectivity of motor, somatosensory, and vestibular areas of the brain^9^, brain activation changes in motor regions under dual tasking conditions^10^, and functional deterioration of gait and balance^11^. Many of these changes appear to reflect neuroplasticity. For instance, individuals with the largest increase in functional connectivity strength between left M1 and right post-central gyrus/superior parietal lobule presented with the least impairments in balance performance changes from pre to post HDBR^9^.

The mechanisms underlying these brain changes resulting from spaceflight and HDBR are not fully understood. Functional and dysfunctional neuroplastic adaptations to the environment^12^, changes in cerebrospinal fluid production and resorption^13^, and focal shifts in bodily fluid^14^ could all contribute to changes observed with MRI. Headward fluid shift occurs in space because gravity no longer pulls fluid to the lower extremities, thus resulting in a more even distribution throughout the body^15^. In HDBR, having the head tilted below the feet also results in fluid shifts towards the head because of a change in the gravitational vector direction^6^. It has been hypothesized that these fluid shifts lead to increased intracranial pressure^16, 17^, as well as redistribution of fluids within the skull^18^.

Alternatively, or in addition, brain changes with spaceflight and HDBR may reflect sensorimotor adaptation. As previously mentioned, HDBR studies have reported that gait and balance declines with HDBR are related to brain structural changes^5, 7, 8^ and functional connectivity changes^9^. Moreover, a case study of one astronaut reported spaceflight-induced changes in resting state functional connectivity between the motor cortex and the cerebellum^4^. It has also been shown in rodent models that spaceflight leads to brain structural changes in sensory and motor brain regions^19^ and the cerebellum^20^.

To facilitate the understanding of brain structural changes with spaceflight and their functional consequences it is important to better characterize the relation between brain tissue changes and fluid shifts. Elucidating the brain changes that occur with HDBR can increase our understanding of the mechanisms underlying motor deficits induced by spaceflight. Diffusion MRI is an MRI sequence that allows quantitative investigation of brain tissue microstructure on the basis of diffusion of water molecules. A novel post-processing technique has been developed that can quantify ‘free water’, which is defined as water molecules that are not hindered or restricted by their surroundings throughout the diffusion experiment^21^. Free water is found in the ventricles, around the brain parenchyma, and in the extracellular space. Free water analysis is therefore an excellent tool to non-invasively investigate cerebral fluid shifts that occur over the course of HDBR, and separate it from possible microstructural changes. For example, the approach has been used to track accumulating free water with substantia nigra degeneration in patients with Parkinson’s disease^22–24^. Furthermore, free water processing estimates conventional Diffusion Tensor Imaging (DTI) metrics adjusted for free water, allowing the analysis of changes in white matter tracts that are unrelated to fluid shifts.

To investigate the effects of HDBR on free water distribution, brain white matter microstructure, and their relation to gait and balance changes, we conducted a prospective longitudinal 70-day 6° HDBR study with 16 male subjects. Diffusion MRI scans and motor behavioral data were collected at 7 time points pre-, during and post HDBR. Outcomes were compared to those of a group of 12 male control subjects who were assessed at 4 time points over a similar time interval. We hypothesized that HDBR would result in gradual fluid shifts from pre to post-intervention. We also predicted that changes in white matter microstructure would correlate with motor behavioral changes induced by HDBR. These effects could either reflect degeneration or adaptive neuroplastic changes depending on the direction of associations with behavior.

## Materials and Methods

### Study design

**Figure 1:**
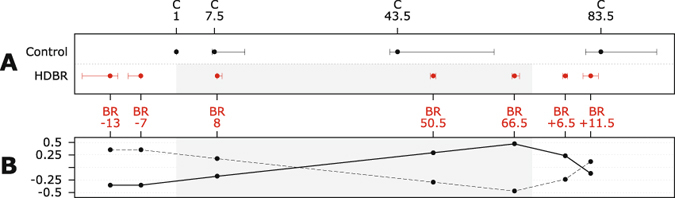
Testing Timeline and Analysis Contrast. (**A**) Testing Timeline: Median (with interquartile range) days at which data was collected for the control (n = 12) and head down-tilt bed rest (HDBR) subjects (n = 16). The gray rectangle in the figures indicates time in HDBR. BR − 13 = 13 days pre-HDBR. BR + 6.5 = 6.5 days post-HDBR. (**B**) HDBR related increases and decreases in FW/FA followed by recovery were modeled separately using the two depicted *a priori* defined contrasts. Y-axes indicate the contrast values used in our analyses that model accumulating effects of HDBR and recovery post-HDBR. (HD)BR = head down-tilt bed rest; solid line = contrast assuming stable outcome measures pre-HDBR, linear increase over HDBR, and partial recovery post-HDBR; dashed line = contrast assuming stable outcome measures pre-HDBR, linear decrease over HDBR, and partial recovery post-HDBR.

The full protocol of this prospective longitudinal study has been published previously^25^. 18 male subjects participated in 6°-HDBR for 70 consecutive days. Previously, we reported on behavioral changes^11^ and functional brain changes^11^ in these subjects. Subjects were randomized to a HDBR exercise group or a HDBR control group (see under *Exercise Intervention*). MRI data were collected for all subjects at 7 time points: pre- (−13 and −7 days), during (8, 50.5 and 66.5 days) and post- (+6.5 and +11.5 days) HDBR. Gait and balance measures were obtained pre- (−13 and −7 days) and post- (+0, +6.5 and +11.5 days) HDBR (see Fig. 1A). The experiment was conducted at the bed rest facility located at the University of Texas Medical Branch (Galveston, TX). Subjects were admitted 13–22 days before going into HDBR and were released 12 days post-HDBR.

We included a group of 16 control subjects from a separate longitudinal study as a reference group for our HDBR subjects. These subjects were recruited from the NASA Johnson Space Center (JSC) Test Subject Facility. They completed the same measurements as the bed rest subjects at four different time points over 90 days (i.e., day 1, 7.5, 43.5, and 83.5; see Fig. 1A). They otherwise went about their daily lives during the study time period.

### Subjects

Two out of the 18 HDBR subjects were excluded from this study because of missing baseline MRI data. The remaining 16 male HDBR subjects were on average 30.4 ± 4.4 years of age at time of admission (range: 25.7–38.9 years). To ensure comparability between control and HDBR subjects, 4 out of 16 control subjects were excluded because they were females while all HDBR subjects were males. The remaining 12 control subjects were on average 41.4 ± 9.9 years of age at time of first assessment (range 26.2–59.7).

All subjects passed an Air Force Class III equivalent physical examination. HDBR subjects were significantly younger (β_age_ = 3.1 ± 1.0; *p* = 0.005) and heavier (β_weight_ = 10.5 ± 4.5; *p* = 0.026) than control subjects. Weight differences were no longer significant after adjusting for age. HDBR and control subjects did not significantly differ in height, body mass index, or systolic blood pressure (*p* > 0.05; data not reported).

This study was approved by the institutional review boards of NASA-JSC, the University of Texas - Medical Branch (UTMB), and the University of Michigan, and conducted in accordance with the Declaration of Helsinki. All subjects provided written informed consent. All subjects received monetary compensation for their participation.

#### Bed rest intervention

HDBR subjects remained in a 6-degree head down tilt position at all times with the exception of 30 minutes during each meal (3 meals per day), when they were allowed to prop up their head with their hand while eating and during the MRI session in which they were in horizontal position. Furthermore, 8 out of the 13 subjects participating in the exercise protocol performed rowing exercises in which the upper body was upright (see under *Exercise Intervention*). In case subjects needed to use the toilet, a bedpan was used to ensure that subjects would not have to get out of head down tilt orientation. Showering was also done in supine position. HDBR subjects were continuously medically monitored. Caloric intake was controlled to maintain constant body weight throughout the HDBR period.

#### Exercise intervention

The current study was embedded in the NASA bed rest framework at UTMB Galveston that combines HDBR experiments from several investigators. Therefore a few of our subjects simultaneously participated in another HDBR supine orientation exercise intervention study^26^. As part of a larger study, our 18 HDBR subjects were randomly assigned to one of three groups: 1) a non-exercise bed rest control group (n = 5); 2) a regular aerobic and resistance exercise group (n = 5); and 3) a flywheel exercise group (n = 8). The exercise protocols for groups 2 and 3 have been described previously^27^. Both HDBR exercise groups performed the same exercise prescription with the same intensity, consisting of 6 days per week of aerobic exercise and 3 days per week of resistance exercise. Aerobic exercise sessions for group 2 consisted of alternating days of continuous cycle exercise for 30 min at 75% of VO_2_peak (maximum rate of oxygen consumption) (3 days/week) with interval treadmill sessions of 30 s, 2 min, or 4 min intervals (3 days/week) at nearly maximal intensity. For group 3 the aerobic exercise was completed using a compact flywheel rowing device for 30 min at 75% VO_2_max (6 days/week). Resistance exercise was performed by both exercise groups every other day and consisted of 3 sets of each of four supine lifts (squat, leg press, unilateral leg curl, and heel raise). Familiarization with the exercise protocol started on average 22 days before HDBR. Full intensity exercise started with the start of HDBR. We statistically controlled for exercise group assignment to have optimal power to detect effects of HDBR on brain white matter.

### Procedure

#### Image acquisition

Diffusion MRIs were acquired on a 3 T Siemens scanner using the following protocol: diffusion-weighted 2D echo-planar imaging sequence (repetition time (TR) = 10100 ms, echo time (TE) = 95 ms, flip angle = 90°, field-of-view (FOV) = 24 × 24 cm, matrix = 128 × 128 slice thickness = 2 mm, 75 contiguous slices (no slice gap), resulting in a voxel size of 1.875 × 1.875 × 2 mm. The b-value was 1,000 s/mm^2^ in 30 non-collinear directions. Each direction was measured twice and two additional volumes without diffusion weighting (b-value = 0 s/mm^2^) were acquired at the beginning and the middle of the sequence. Acquisition time was ~10 min.

In 18 scans, the scan parameters were unintentionally slightly altered. For 8 scans additional slices were collected (79–87) and for these scans the TR was increased (10200–10700). For 7 other scans the FOV was smaller (n = 3, FOV = 220) or larger (n = 4, FOV = 250/270). Finally, TR was increased in 2 scans (10200 and 10700) and the number of slices was increased in 1 scan (79) without other parameter changes. These alterations were not systematically distributed over the time points regarding the intervention and recovery time course. Removing these scans did not change the pattern of our results. Unfortunately, DTI baseline data was not collected for 2 of our 5 control subjects. These subjects were not included in our analyses. Complete data for 7 time points was available for 12 of the remaining 16 HDBR subjects. Of the remaining 4 subjects data was available for 6 time points. Data was missing because of either loss during transfer (n = 1), it had not been collected because of time constraints (n = 1), or large differences in the acquired slice thickness (3 mm/4 mm; n = 2). Complete data for 4 time points was available for 8 of the 12 control subjects. For two control subjects, data from 3 time points was available and for two other control subjects, data from 2 time points was available. Reasons for missing data were time constraints (n = 3) and scan artifacts (n = 3).

#### Image processing

The following software packages were used for image analysis: FMRIB Software Library (FSL) version 5.0.8; Statistical Parametric Mapping (SPM8, v4667); Statistical Parametric Mapping (SPM12, v6685); Advanced Normalization Tools (ANTs 1.9.x,^28, 29^); and Matlab (MATLAB 8.6.0.267246 - R2015b) code for ‘free water’ analysis^21^. We used a stepwise approach that takes into account the longitudinal nature of the data to bring the individual free water and free water adjusted DTI images into MNI152 (Montreal Neurological Institute) standard space. This pipeline is an adaptation of that described by Schwarz *et al*.^30^:

1. Pre-processing: A Rician filter was applied to the Diffusion Weighted Images (DWI) data to remove random noise^31^. Motion correction and eddy current correction were carried out in FSL by registering all DWI images to the average of the two b = 0 images. Subsequently, the diffusion gradients were adjusted for the transformations.
2. Free Water Analysis: All unprocessed DWI images and motion correction plots were inspected for volumes with excessive scan motion or scan artifacts. These volumes were then removed from the data. Subsequently, Matlab code implementing the algorithm in ref. 21 was used to fit the free-water model. The model has two compartments: one compartment has diffusivities fixed to water in body temperature (3*10^−3^ mm^2^/s) that model water molecules that are free to diffuse, The second compartment models the remaining diffusion in the tissue using a diffusion tensor^21^. This resulted in the following metrics per subject per time point: (i) fractional anisotropy (FA) images adjusted for free water; the unitless FA ranges from 0–1 and indicates the preferred directionality of water diffusion in tissue. In tightly packed fiber bundles, such as white matter, FA tends to be higher than in non-structured tissues. (ii) free water images (FW). This parameter maps the fractional volume of the free-water compartment with a range of 0–1, where 1 is found in voxels that are completely filled with free-water, such as in the ventricles and around the brain parenchyma. Thus, the volume of FW per voxel can be obtained by multiplying the FW value by the cubic size of the voxel. FW represents an index of water molecules that are not restricted or hindered by their surroundings^21^.
3. Normalization: For normalization we followed the longitudinal image registration pipeline described by Schwarz *et al*.^30^, which boosts sensitivity and specificity of subsequent voxel-based analyses such that it is favorable over tract-based spatial statistics. In short, for each subject, a subject-specific template was built using ANTs’ buildtemplateparallel. sh script. In a second step, these subject-specific templates were warped to MNI152 common space using ANTs’ SyN algorithm. In a third step, we combined the linear and non-linear warp parameters from the individual subject FA image onto the subject specific FA template and onto MNI152 common space into one flow field. These flow fields were then used to normalize the FA (adjusted for free water) and FW images into MNI152 common space.
4. Smoothing: The normalized images were smoothed to increase signal to noise ratio with a Gaussian kernel that had a standard deviation of 3.4 mm, equivalent to ~8 mm full-width at half-maximum.

#### Reliability of diffusion MRI metrics

Fluctuations of the MR signal such as random noise and scanner drift can adversely affect the power to detect a true effect in a longitudinal study. To assess the longitudinal reliability of our diffusion weighted sequence we used the intra-class correlation coefficient (ICC) toolbox for SPM^32^ in combination with SPM8 to evaluate whether there is a consistent agreement^33^ of the FA and FW maps from (a) 13 days pre-HDBR to 7-days pre-HDBR; (b) from assessment day 1 to assessment day 7.5 in the control subjects, and (c) from assessment day 1 to assessment day 83.5 in the control subjects. The ICC toolbox for SPM provides voxel-wise ICC maps and a summary ICC measure that can range from 0 (no agreement) to 100 (perfect agreement).

#### Functional Mobility and Balance Assessments

The description of functional mobility and balance tests and their change over the course of bed rest have been described previously^9, 11, 25^.

Locomotion was assessed using the Functional Mobility Test; an obstacle course that is partly set up on a concrete floor (1^st^ half) and partly on a medium density foam floor (2^nd^ half) to increase the balance challenge on this part of the test. The outcome measure of the test was completion time in seconds of the 1^st^ half, the 2^nd^ half, and the total course.

Balance was assessed using Sensory Organization Tests (SOT) (i.e., a computerized dynamic posturography system) provided by the EquiTest System platform (NeuroCom, Clackamas, OR)^3^. During testing, subjects were instructed to maintain stable upright posture for three, 20-second trials with eyes closed. All trials were conducted with sway-referenced support surface intended to disrupt somatosensory feedback and therefore reflect how well vestibular inputs could be utilized to maintain balance (referred to as SOT-5). In addition to SOT-5, subjects completed the task once more while they were tasked to pitch their heads ± 20° at 0.33 Hz as cued by an oscillating tone provided over headphones (referred to as SOT-5M). SOT-5M is more difficult than the SOT-5 by requiring voluntary head movements and thus integration of both semicircular canal (angular) and otolith (linear) cues.

### Voxel-Wise Data Analysis

#### Masking

Voxel-wise analyses of FA was conducted within a white matter mask. This mask was constructed by binarizing the mean FA image (in MNI space) of all subjects thresholded at 0.2 (the typical cut off value for white matter). From this image all voxels that were present in less than 50% of the images were then excluded to form the final mask^30^. FW analyses were conducted within a mask that covered the entire brain and which was constructed by applying FSL’s brain extraction tool to the FMRIB58_FA_1 mm standard space image.

#### Changes in FA and FW during HDBR and versus control subjects

To evaluate brain changes during HDBR we calculated the regression slope for HDBR subjects, from pre-HDBR to the last day in HDBR. To calculate this slope, the last image collected pre-HDBR was treated as if it were collected immediately prior to HDBR. This analysis assumes linear changes over time during HDBR within the HDBR group. Therefore, post-HDBR images are not included because any recovery after HDBR could result in departure from linearity of the slope. We also calculated the slope of the control subjects (over all 4 time points). The resulting slope images of HDBR and controls were then compared using a two-sample t-test. To aid the interpretation of any potential group by time interaction we also separately tested the slope of the control group using a one-sample t-test. Because of the recent concern about inflation of false positives for cluster-wise inference with parametric models^34^ we used non-parametric permutation tests with threshold free cluster enhancement (TFCE) controlled for the family-wise error (FWE) rate (*p* < 0.05) for inference (FSL’s Randomise^35^). All analyses were conducted running 15,000 random permutations, adjusted for age.

#### Changes in FA and FW: pre- to post-HDBR

To test for any focal changes in FA or FW from pre- to post-HDBR including data from all time points we used a linear mixed model^36^. We applied a pre-defined contrast to test for accumulating focal changes in FA or FW during HDBR followed by recovery post-HDBR (see Fig. 1B). By using this contrast the imaging data of each assessment time point is multiplied by a weighting factor. The statistical analyses test if overall the subjects’ sum images (of these weighted images) contain regions that are significantly different from zero. Our contrast therefore uses data from all subjects and all assessment time points. Such a linear mixed-model contrast cannot directly be implemented in Randomise and we therefore used SPM12 for inference. Additionally, we conducted posthoc tests on those voxels that showed a statistical agreement with the pre-defined contrasts, to compare FA or FW at time points during- and post-HDBR to the average pre-HDBR to explore the time course of changes over HDBR and recovery.

These analyses were adjusted for exercise intervention group assignment by entering HDBR group (regular exercise, flywheel exercise, and non-exercise) and the group-by-time interaction terms into our model (as outlined in ref. 37). This way, we adjusted for the main effects of group as well as the group-by time interaction. All analyses were FWE corrected (*p* < 0.05).

To test for overall volume changes in FW, for each subject we obtained the net FW change over all voxels from pre-HDBR to each time point during and to post-HDBR. We used one-sample t-test to test whether there was a global change from pre-HDBR at any given time point. These analyses were Bonferroni corrected (peak-level; *p* < 0.05).

#### Brain-behavior relationships

We recently reported that HDBR results in significant deterioration in functional mobility and balance^11^. These data were collected during the same bed rest study as the current one, from the same subjects. Here, we test whether these sensorimotor changes correlate with any changes in FA or FW. For this we calculated new contrast images by subtracting FA or FW maps from HDBR -7 from HDBR 66.5. We then tested which brain regions significantly correlated with the score difference in FMT or SOT performance from 7 days pre-HDBR to immediately post-HDBR (HDBR + 0). Brain regions were restricted to those areas in which we observed changes from pre to post-HDBR in each diffusion MRI metric respectively.

Additionally, we created a Region of Interest (ROI) from an area in which we previously observed a significant association between changes in gray matter volume and SOT5 performance (*unpublished data*). This ROI was then used as a mask to test for the associations between FW changes and FMT/SOT changes.

Finally, we tested for associations between changes in SOT and FMT performance and FW changes in 4 pre-selected ROIs, i.e.: the left pre- and post-central gyrus (obtained from the International Consortium for Brain Mapping (ICBM) atlas^38^), right cerebellum lobule V (obtained from the SUIT atlas^39^), and a right vestibular cortex spherical ROI (defined according to refs 40, 41). These ROIs were previously used to test the association between motor behavioral changes and gray matter changes from pre- to post-HDBR (*unpublished data*). All analyses were Bonferroni corrected (*p* < 0.05).

## Results

### Reliability of diffusion MRI metrics

Overall, the median whole-brain ICC value for all groups, time intervals and diffusion MRI metrics ranged from 70.0 to 83.6, indicating good to excellent reliability. For FA and FW, the median whole brain ICC value over all voxels was: a) 71.1 and 81.3 from 13 to 7 days pre-HDBR; b) 71.8 and 83.6 from 0 to 7.5 days for control subjects; and c) 70.0 and 81.7 from 0 to 83.5 days for control subjects.

### Changes in FA and FW during HDBR and versus control subjects

**Figure 2:**
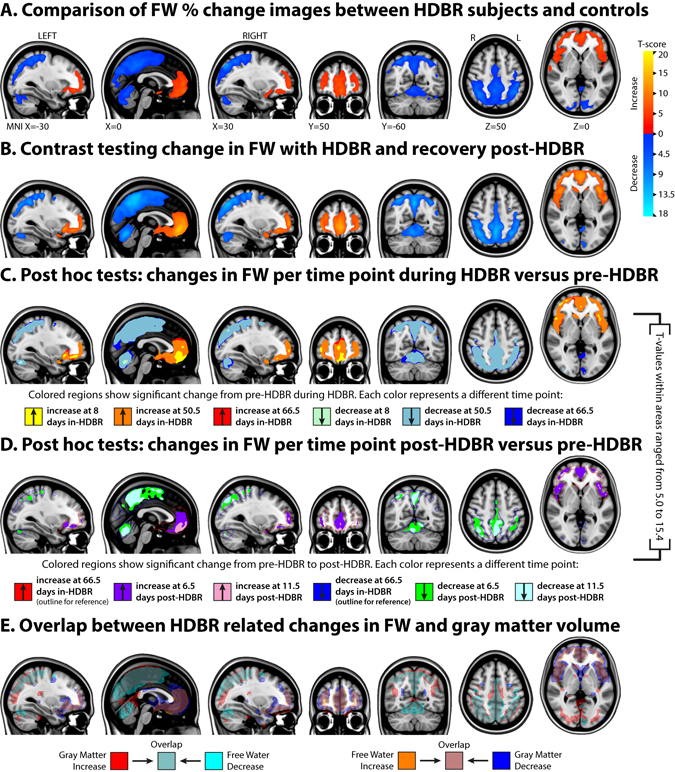
Effects of HDBR on Free Water distribution. Group level results of changes in FW are overlaid on the MNI152 common template. (**A**) Results from a paired-sample t-test comparing slope images of linear changes from pre-HDBR to the end of HDBR relative to changes over a similar time course in control subjects. Blue-to-light blue and red-to-yellow gradients show regions in which HDBR subjects show significantly larger FW decrease and increase respectively relative to control subjects. (**B**) Changes within HDBR subjects over all 7 time points following the linear mixed model contrast specified in Fig. 1B; (**C**,**D**) Results from post-hoc pairwise tests comparing pre-HDBR with time points during HDBR (**C**) and with post-HDBR (**D**). Each color indicates at which time point certain increases/decreases appeared first. The Regions that show significant increase and decrease early in HDBR expand in size with accumulating time in HDBR and diminish in size post-HDBR. Note: the red and blue outlines in (**D**) are actually the maximum effects of HDBR at 66.5 days in HDBR and serve as reference for the interpretation of recovery. (**E**) Overlap between regions with changes in Free Water and Gray Matter Volume (*unpublished data*) with HDBR; Coordinates indicate MNI152 space coordinates; all analyses are FWE corrected.

FA and FW in the control subjects did not significantly change over time (i.e., the slope was not significantly different from 0). No significant differences were observed when comparing the slopes of FA changes over time between HDBR and control subjects. Although not significant, the change in FA in the voxel with the smallest p-value showing FA increase was at MNI −17, −81,29. The pre-HDBR FA group mean value of this voxel was 0.38 (se = 0.07) and showed a daily FA increase adjusted for age and relative to control subjects of 0.10% (se = 0.03). For regions showing FA decrease the peak MNI voxel was −16, −26, −14 (pre-HDBR group mean FA = 0.57, se = 0.12) and its corresponding adjusted daily FA decrease was 0.08% (se = 0.02). Comparing the slopes of FW changes between HDBR and control subjects showed a significant increase in FW in HDBR around the frontal poles, frontal operculum, medial and orbital frontal cortex, and the inferior frontal gyrus. Significant decrease in FW in HDBR was found in the lateral occipital cortex, superior parietal lobule, and the cerebellum (see Fig. 2A and Supplementary Tables).

### Changes in FA and FW: Pre to post HDBR

Applying a pre-defined contrast that assumes a stable baseline from 13 to 7 days pre-HDBR followed by either linear increase or decrease with HDBR and then recovery post-HDBR we observed significant widespread extensive changes in FW (see Fig. 2B). These FW changes overlap extensively with regions showing group differences in change over time (Fig. 2A). We did not observe any changes in global FW volume from pre-HDBR at any time point during or post-HDBR. No FA changes corresponding with the pre-defined contrasts were observed. Regions in which there was FW decrease followed by recovery post-HDBR covered the bilateral anterior and superior posterior lobes of the cerebellum, the post-central gyrus, the precuneus, the superior frontal gyrus and the superior parietal gyrus (see Supplementary Tables for an overview of all regions). Regions that showed FW increases followed by recovery post-HDBR were observed around the falx cerebri in the frontal lobe, in the insula, around the hypothalamus, in the inferior frontal gyrus, and the orbitofrontal gyrus.

**Figure 3:**
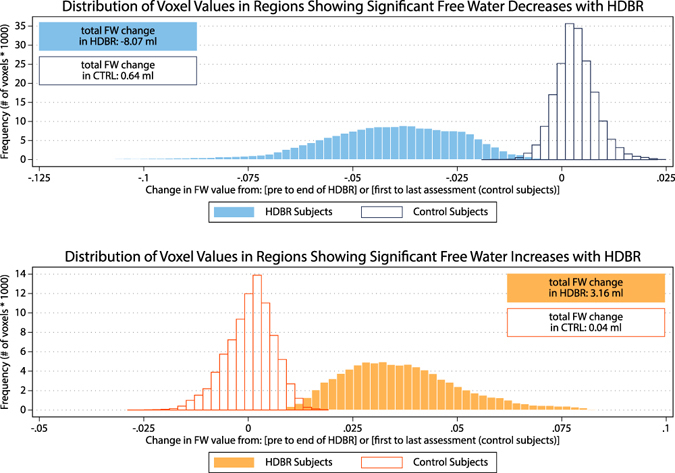
Magnitude of FW change with HDBR. The top graph shows the distribution of values of FW change for all voxels in the brain region in which the significant group-by-time interaction indicated a decrease in FW in HDBR (see Fig. 2A, blue regions). The bottom graph shows the distribution of values of FW change for all voxels in the brain region in which the significant group-by-time interaction indicated an increase in FW in HDBR (see Fig. 2A, red regions).

Posthoc pairwise comparisons of all consecutive time points during and post-HDBR with the pre-HDBR baseline revealed areas with gradual increases in FW and areas with gradual decreases in FW up until 50.5 days in HDBR after which the gradual changes stabilized (see Fig. 2C,D). Post-HDBR the FW changes recovered, but had not yet fully cleared up at 11.5 days post-HDBR. To obtain an understanding of the magnitude of FW changes we calculated the total volume of FW change over all voxels in the locations in which we found significant association with the pre-defined contrasts (Fig. 2A). FW change for HDBR subjects was defined as the difference in FW from 7 days pre-HDBR to 66.5 days in HDBR. The FW change for control subjects was defined as the difference between the first and the last assessment time point. Voxel values were obtained from the averaged difference maps and were derived separately for regions showing significant increases and significant decreases with HDBR. Over all voxels in regions showing significant differences in slopes, HDBR subjects lost on average 8.1 ml of FW in the posterior parietal region and gained on average 3.2 ml in the fronto-temporal area (see Fig. 3). FW volume changes in control subjects were on average 0.5 ml or less in both areas. The average FW value within the posterior-parietal area changed from 0.36 to 0.32 in HDBR subjects (pre-HDBR to last assessment in HDBR) and remained 0.38 in CTRL subjects (first to last assessment). In the fronto-temporal cluster, these values were 0.37 to 0.41 and 0.38 respectively.

### Brain-behavior relationships

We tested for associations between changes in FW with changes in FMT, SOT-5, and SOT-5M performance from 7 days pre-HDBR to 66.5 days in HDBR. We did not test for correlations with FA changes because we did not observe significant changes in FA over HDBR. All brain-behavior analyses were restricted to those areas in which change with HDBR was observed for the particular diffusion MRI metric. No correlations between FMT and FW changes were observed.

**Figure 4:**
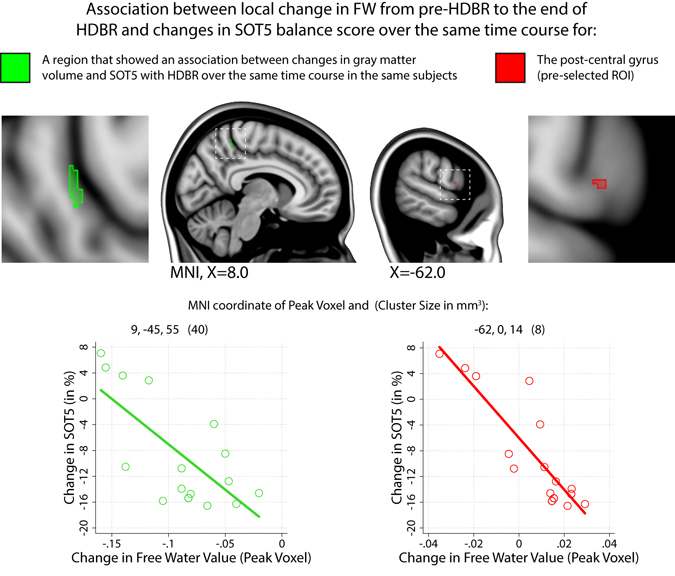
Correlations between changes from pre-HDBR to the end of HDBR in Free Water and Balance. Change in balance performance (SOT5) was negatively correlated with FW changes in two predefined regions of interest: 1) a region covering the precuneus (green accents); and 2) the lateral post-central gyrus (red accents); Graphs show the direction of the association. I.e., decreases in FW result in smaller balance decrements or even improvements from pre-HDBR to the end of HDBR whereas larger FW increases result in larger balance deterioration. SOT5 = Sensory Organization Test 5; MNI = Montreal Neurological Institute. All results are corrected for multiple comparisons.

We observed a significant (FWE corrected) negative association between balance control (SOT5) changes and FW changes with HDBR within the precuneus/post-central gyrus (see Fig. 4; green colors). Thus, the larger the decrease in FW, the smaller the decrement (with some even showing increment) in balance performance from pre-HDBR to the end of HDBR. In a previous analysis we observed that larger increases in gray matter volume of this region with HDBR correlated with smaller decrements or even improvements of balance performance (*unpublished data*).

Within our post-central gyrus ROI, on the lateral border with the pre-central gyrus, we observed a significant (FWE corrected) negative association between changes in FW and changes in balance performance (SOT5) (see Fig. 4; red colors). Subjects exhibiting a larger decrease in FW showed a smaller decrease (or even an increase) in balance performance from pre-HDBR to the end of HDBR. No associations were observed between FMT, SOT-5, or SOT-5M and FW changes in other predefined ROIs.

### Similarities with volumetric gray matter changes induced by HDBR and spaceflight

In a previous publication we reported on gray matter changes over the course of the same HDBR study as we report on here (*unpublished data*). In addition, we also investigated gray matter changes in astronauts from pre- to post-flight^5^. To demonstrate the similarities in the patterns of gray matter changes in these two studies with the pattern of FW changes that we report here, we have registered these results to the same common template (see Fig. 2E and Supplementary Figure 1). These qualitative comparisons reveal striking similarities in the pattern of FW changes over the course of HDBR with changes in GM over the course of HDBR and from pre- to post-spaceflight. Areas that show gray matter increase with HDBR and spaceflight largely overlap with FW decreases in HDBR and vice versa.

## Discussion

We investigated changes in white matter microstructure and white and gray matter free water as a function of long-duration HDBR in 16 subjects. We tested these changes against the course of changes over a similar time interval in a sample of 12 control subjects. In line with our hypotheses, we found major intracranial fluid (i.e., FW) shifts that increased in size with increasing time in HDBR and largely recovered at ~12 days post-HDBR.

In contrast to what we hypothesized, white matter microstructure was not affected by HDBR. Larger decreases in FW in the precuneus, post-central gyrus and motor cortex were associated with smaller decrements (or even improvements) in balance performance over the course of HDBR. The pattern of FW changes was strikingly similar to the pattern of gray matter changes that we^5^ and others^7, 8^ have previously reported as a function of HDBR and spaceflight^5^.

These results provide important new insights into brain structural alterations in HDBR that could explain functional^9, 10^ and behavioral changes^11^ with HDBR, although these processes may be unrelated or separately caused by a common underlying factor. HDBR is commonly used to induce headward fluid shifts in order to investigate microgravity-related brain and ocular changes on Earth^6^. Previous spaceflight studies have reported deterioration of visual acuity that was linked to spaceflight induced ocular structural changes^42–44^. HDBR studies have reported related ocular structural changes^45, 46^. These structural changes are thought to result from increased intracranial pressure due to headward fluid shifts in microgravity and HDBR^14^. An anatomical MRI study in 7 subjects comparing the focal distribution of subarachnoidal cerebrospinal fluid (CSF) in prone, supine, and lateral positions showed that CSF volume decreases on the side of the head closest to the ground due to downward brain movement with gravity^18^. Additionally, a T2 MRI study in 5 subjects showed that 40 minutes of head down-tilt resulted in less water in a midsagittal superior subarachnoidal ROI^47^. In the latter study, no fronto-temporal ROIs were investigated, so it remains unclear if there were any focal increases in CSF. Our results corroborate these previous studies, and add that these fluid changes are not associated with microstructural changes of the white matter.

By using a control group and by having multiple assessments pre-, during, and post-HDBR we were able to investigate the time course of the fluid redistribution and its recovery. FW shifts present as early as 8 days in HDBR and continue to expand in size until 50.5 days in HDBR after which it seems to plateau. Post HDBR there is a steep recovery timecourse, although 11.5 days post-HDBR the fluid shift has not fully recovered. Thus, while previous T1^18^ and T2^47^ studies showed almost immediate fluid shifts with posture change, we find that FW continues to accumulate for at least another 42.5 days while in HDBR. The pattern of FW changes over time is very similar to that of gray matter volume changes that we observed in anatomical MRI analysis of these subjects^5^. It is therefore likely that the HDBR-induced gray matter changes that we and others^7, 8^ observed at least partially reflect fluid redistribution, rather than neuronal atrophy, dendritic branching, or other factors thought to contribute to gray matter changes. We note that a complete separation between fluid redistribution and plastic gray matter changes, using the MRI techniques available, is not possible, since fluid redistribution affects the segmentation approaches that estimate brain volume^48^. On the other hand gray matter degeneration increases the extracellular spaces and free-water. However, identifying that fluid redistribution may explain the gray matter changes, which were previously observed, is important since neuronal atrophy causing plastic gray matter changes is less likely to be reversible, whereas fluid redistribution can change within short time periods^18^. The fact that we observe recovery in most gray matter areas further suggests that fluid redistribution may at least partially underlie the previously observed gray matter changes with HDBR.

It has been suggested that spaceflight and HDBR could result in a decrease in venous outflow and an increase in intracranial CSF volume resulting in overall increased intracranial pressure^49, 50^. Our study does not show indications for an overall increase in fluid within the intracranial compartment, but rather a *redistribution* of existing fluids. Intracranial pressure in the supine position is normally measured at the foramen of Monro^16^. Potentially, changes in intracranial pressure with HDBR and spaceflight differ per region. Perhaps, in addition to the focal increases in pressure, there are also focal pressure decreases that arise with HDBR and spaceflight. Furthermore, a study in rabbits showed that increases in intracranial pressure emerged almost immediately after head down-tilt, but reverted back close to baseline within 8 hours^50^. This suggests that fluid redistribution and intracranial pressure changes may be independent processes to a certain extent.

The magnitude of FW changes was substantial; within the posterior parietal region showing FW decrease with HDBR the average change per voxel was 11.1%; within the fronto-temporal region showing FW increases with HDBR the average change per voxel was 10.8%. Although a global CSF volume increase can affect motor behavior^51, 52^, few regions in which we observed FW changes were associated with motor behavioral changes. Subjects who presented with larger decreases in FW in the right precuneus / post-central gyrus and left post-central gyrus exhibited smaller decrements or even improvements in standing balance performance with HDBR. This is an interesting finding in light of our previous work in which we reported volumetric gray matter increases in these regions that also correlated with more favorable balance performance post-HDBR (*unpublished data*). Together, the regional co-occurrence of gray matter increases and FW decreases that both correlate with balance performance add to the idea that HDBR results in adaptive structural changes (e.g., dendritic branching^53^). The medial portion of the primary somatosensory cortex receives inputs from the thigh, leg and foot, while the precuneus is involved in orientation in space and time^54^. HDBR and spaceflight environments are different from Earth, both resulting in muscle unloading and sensory reweighting with adaptation of movements to the new environment^55^. Sensorimotor adaptation is associated with structural plasticity in primary motor circuits^56^ and functional brain changes of motor networks^57^. Furthermore, changes in the sensory system have a fundamental role in sensorimotor adaptation, including reweighting of sensory inputs^58^. Thus, the FW changes in the precuneus and post-central gyrus that we observed could reflect reintegration of sensory inputs for adaptation to the axial unloading and immobility in the HDBR environment. Lee and Whitt have also shown that when sensory inputs are reduced—such as occurs with somatosensory inputs in HDBR—sensory processing is up-regulated in the affected primary sensory cortex^59^. FW decreases and GM volume increases in somatosensory cortices with HDBR may reflect this process.

The FW changes that we observed did not fully recover at 12 days post-HDBR. Therefore, it is yet unknown if any of these FW changes are permanent or resolve at longer times post-HDBR. Studies with longer follow-up will be necessary to further determine the course of FW changes after HDBR.

Our results do not suggest widespread detrimental effects on white matter fibers. Rather, they indicate fluid redistribution within gray matter tissue that largely recover within two weeks after cessation of HDBR. As has been discussed above, decreased FW in the post-central gyrus and precuneus were associated with smaller decrements in balance, which was likely related to adaptive structural gray matter changes. Thus, HDBR as a spaceflight analog does not indicate that a microgravity environment poses excessive risk for human brain white matter microstructure. However, astronaut studies are needed to validate these findings. One other study previously looked at changes in white matter microstructure with HDBR and reported that 30 days of HDBR resulted in widespread FA changes^7^. However, these results were not adjusted for free water and thus likely reflect water redistribution. Moreover, these results were not adjusted for multiple comparisons and the uncorrected p-value was set to be very liberal at 0.01, making the generalizability of these findings difficult.

Our results are also reassuring for the large community of bedridden individuals, such as those residing in nursing homes and individuals after major surgery. Long duration bed rest in healthy older adults has been associated with functional disability^60^ and physical fitness^61^. Despite fluid shifts related to the supine body orientation, which seem reversible, our results do not suggest that long duration bed rest results in irreversible brain changes.

A major strength of our study is the direct comparison of control subjects and HDBR subjects, which previous HDBR MRI studies have not done. Furthermore, we assessed both control and HDBR subjects at multiple time points. This allowed us to interpret the temporal dynamics of brain changes and recovery. In addition, by using the free-water analysis^21^ we were able to tease apart fluid shifts from changes in microstructure. This approach has substantially improved the understanding of previous reports on structural brain changes following HDBR. Moreover, all analyses in the current study were corrected for multiple comparisons, thereby reducing the chance of type I errors. In addition, ICC analysis showed that the reproducibility of our normalized FW and FA images that resulted from our MR sequence and processing pipeline was very good and similar or better than what has been reported for these metrics previously^62^.

Although HDBR subjects were assessed at seven different time points we did not include measurements immediately after the start of HDBR. Therefore, we were unable to monitor the time course of near instant fluid shifts induced by posture change. Future studies will need to include such an assessment time point. Likewise, longer follow-up is necessary to determine whether HDBR causes permanent effects on FW distribution. The fact that we did not identify microstructural white matter damage does not mean HDBR does not affect white matter microstructure. Identifying subtle white matter changes may require a larger sample size, or longer duration of HDBR. Furthermore, although we adjusted for exercise, residual confounding may have affected our ability to detect a true effect of HDBR on white matter microstructure.

70 Days of HDBR can result in a redistribution of subarachnoid and interstitial fluid without an overall volume change that largely recovers two weeks after HDBR. At the same time, HDBR does not seem to affect the microstructure of the white matter of the brain. There is a large overlap in the distribution of FW increases with gray matter volume decreases and vice versa, which corroborate intracranial fluid redistribution as an interpretation. FW changes in the post-central gyrus and precuneus correlated with changes in balance control with HDBR and could therefore reflect adaptive structural neuroplasticity. Future studies are warranted to determine causality and underlying mechanisms. Our study provides novel insights into brain structural changes in HDBR, which could potentially extrapolate to astronauts after spaceflight. Replication in independent samples is warranted to validate our results in HDBR and with spaceflight.

## Electronic supplementary material

Supplementary Figure and Tables

## References

[1] Mulavara AP (2010). Locomotor function after long-duration space flight: effects and motor learning during recovery. Experimental brain research.

[2] Mulavara AP (2012). Vestibular-somatosensory convergence in head movement control during locomotion after long-duration space flight. Journal of vestibular research: equilibrium & orientation.

[3] Wood SJ, Paloski WH, Clark JB (2015). Assessing Sensorimotor Function Following ISS with Computerized Dynamic Posturography. Aerospace medicine and human performance.

[4] Demertzi A (2016). Cortical reorganization in an astronaut’s brain after long-duration spaceflight. Brain structure & function.

[5] Koppelmans, V., Bloomberg, J. J., Mulavara, A. P. & Seidler, R. D. Brain Structural Plasticity With Spaceflight. *npj Microgravity* **2**, doi:10.1038/s41526-016-0001-9 (2016). 10.1038/s41526-016-0001-9 PMC5460234 28649622

[6] Hargens AR, Vico L (2016). Long-duration bed rest as an analog to microgravity. Journal of applied physiology.

[7] Li K (2015). Effect of Simulated Microgravity on Human Brain Gray Matter and White Matter-Evidence from MRI. PloS one.

[8] Roberts DR (2015). Structural Brain Changes following Long-Term 6 degrees Head-Down Tilt Bed Rest as an Analog for Spaceflight. AJNR. American journal of neuroradiology.

[9] Cassady, K. *et al*. Effects of a spaceflight analog environment on brain connectivity and behavior. *NeuroImage*, doi:10.1016/j.neuroimage.2016.07.029 (2016). 10.1016/j.neuroimage.2016.07.029 27423254

[10] Yuan, P. *et al*. Increased brain activation for dual tasking with 70-days head-down bed rest. *Frontiers in systems neuroscience*, doi:10.3389/fnsys.2016.00071 (2016). 10.3389/fnsys.2016.00071 PMC4993791 27601982

[11] Koppelmans V (2015). Exercise as potential countermeasure for the effects of 70 days of bed rest on cognitive and sensorimotor performance. Frontiers in systems neuroscience.

[12] Reschke MF, Bloomberg JJ, Harm DL, Paloski WH (1994). Space flight and neurovestibular adaptation. Journal of clinical pharmacology.

[13] Kramer LA (2015). MR-derived cerebral spinal fluid hydrodynamics as a marker and a risk factor for intracranial hypertension in astronauts exposed to microgravity. Journal of magnetic resonance imaging: JMRI.

[14] Nelson ES, Mulugeta L, Myers JG (2014). Microgravity-induced fluid shift and ophthalmic changes. Life.

[15] Diedrich A, Paranjape SY, Robertson D (2007). Plasma and blood volume in space. The American journal of the medical sciences.

[16] Alperin N, Lee SH, Bagci AM (2015). MRI measurements of intracranial pressure in the upright posture: The effect of the hydrostatic pressure gradient. Journal of magnetic resonance imaging: JMRI.

[17] Steinbach GC, Macias BR, Tanaka K, Yost WT, Hargens AR (2005). Intracranial pressure dynamics assessed by noninvasive ultrasound during 30 days of bed rest. Aviation, space, and environmental medicine.

[18] Bijsterbosch JD (2013). The effect of head orientation on subarachnoid cerebrospinal fluid distribution and its implications for neurophysiological modulation and recording techniques. Physiological measurement.

[19] D’Iachkova L, N. [Ultrastructural changes in somatosensory cortex of albino rats during space flight]. *Izvestiia Akademii nauk. Seriia biologicheskaia/Rossiiskaia akademiia nauk*, 372–375 (2007). 17853701

[20] Holstein GR, Kukielka E, Martinelli GP (1999). Anatomical observations of the rat cerebellar nodulus after 24 hr of spaceflight. Journal of gravitational physiology: a journal of the International Society for Gravitational Physiology.

[21] Pasternak O, Sochen N, Gur Y, Intrator N, Assaf Y (2009). Free water elimination and mapping from diffusion MRI. Magnetic resonance in medicine.

[22] Burciu RG (2016). Free-water and BOLD imaging changes in Parkinson’s disease patients chronically treated with a MAO-B inhibitor. Human brain mapping.

[23] Ofori E (2015). Longitudinal changes in free-water within the substantia nigra of Parkinson’s disease. Brain: a journal of neurology.

[24] Planetta PJ (2016). Free-water imaging in Parkinson’s disease and atypical parkinsonism. Brain: a journal of neurology.

[25] Koppelmans V (2013). Study protocol to examine the effects of spaceflight and a spaceflight analog on neurocognitive performance: extent, longevity, and neural bases. BMC neurology.

[26] Downs M, Buxton R, Goetchius E, DeWitt J, Ploutz-Snyder L (2016). Individual Variability in Aerobic Fitness Adaptations to 70 Days of Bed Rest and Exercise Training: 2448 June 3, 10: 00 AM–10: 15 AM. Medicine and science in sports and exercise.

[27] Ploutz-Snyder LL (2014). Integrated resistance and aerobic exercise protects fitness during bed rest. Medicine and science in sports and exercise.

[28] Avants BB (2010). The optimal template effect in hippocampus studies of diseased populations. NeuroImage.

[29] Avants BB (2011). A reproducible evaluation of ANTs similarity metric performance in brain image registration. NeuroImage.

[30] Schwarz CG (2014). Improved DTI registration allows voxel-based analysis that outperforms tract-based spatial statistics. NeuroImage.

[31] Manjon JV (2013). Diffusion weighted image denoising using overcomplete local PCA. PloS one.

[32] Caceres, A. & Mehta, M. *ICC Toolbox for SPM5*, http://www.kcl.ac.uk/ioppn/depts/neuroimaging/research/imaginganalysis/Software/ICC-Toolbox.aspx (2015).

[33] Shrout PE, Fleiss JL (1979). Intraclass correlations: uses in assessing rater reliability. Psychological bulletin.

[34] Eklund A, Nichols TE, Knutsson H (2016). Cluster failure: Why fMRI inferences for spatial extent have inflated false-positive rates. Proceedings of the National Academy of Sciences of the United States of America.

[35] Winkler AM, Ridgway GR, Webster MA, Smith SM, Nichols TE (2014). Permutation inference for the general linear model. NeuroImage.

[36] Kurth, F., Luders, E. & Gaser, C. *VBM8-Toolbox Manual*, http://dbm.neuro.uni-jena.de/vbm8/VBM8-Manual.pdf (2010).

[37] Gläscher, J. P. & Gitelman, D. *Contrast weights in flexible factorial design with multiple groups of subjects*, https://http://www.researchgate.net/publication/267779738_Contrast_weights_in_flexible_factorial_design_with_multiple_groups_of_subjects (2008).

[38] Mazziotta J (2001). A four-dimensional probabilistic atlas of the human brain. Journal of the American Medical Informatics Association: JAMIA.

[39] Diedrichsen J, Balsters JH, Flavell J, Cussans E, Ramnani N (2009). A probabilistic MR atlas of the human cerebellum. NeuroImage.

[40] Eickhoff SB, Weiss PH, Amunts K, Fink GR, Zilles K (2006). Identifying human parieto-insular vestibular cortex using fMRI and cytoarchitectonic mapping. Human brain mapping.

[41] zu Eulenburg P, Caspers S, Roski C, Eickhoff SB (2012). Meta-analytical definition and functional connectivity of the human vestibular cortex. NeuroImage.

[42] Kramer LA, Sargsyan AE, Hasan KM, Polk JD, Hamilton DR (2012). Orbital and intracranial effects of microgravity: findings at 3-T MR imaging. Radiology.

[43] Mader TH (2011). Optic disc edema, globe flattening, choroidal folds, and hyperopic shifts observed in astronauts after long-duration space flight. Ophthalmology.

[44] Riascos R (2015). Novel finding of optic nerve central T2 hypointensity utilizing 3 Tesla MR imaging. The neuroradiology journal.

[45] Taibbi G (2014). Ocular outcomes evaluation in a 14-day head-down bed rest study. Aviation, space, and environmental medicine.

[46] Taibbi G (2016). Ocular Outcomes Comparison Between 14- and 70-Day Head-Down-Tilt Bed Rest. Investigative ophthalmology & visual science.

[47] Caprihan A, Sanders JA, Cheng HA, Loeppky JA (1999). Effect of head-down tilt on brain water distribution. European journal of applied physiology and occupational physiology.

[48] Eriksson SH (2009). Quantitative grey matter histological measures do not correlate with grey matter probability values from *in vivo* MRI in the temporal lobe. Journal of neuroscience methods.

[49] Alperin N, Lee SH, Sivaramakrishnan A, Hushek SG (2005). Quantifying the effect of posture on intracranial physiology in humans by MRI flow studies. Journal of magnetic resonance imaging: JMRI.

[50] Kawai Y (2003). Effects of Microgravity on Cerebral Hemodynamics. Yonago Acta medica.

[51] Zur D, Naftaliev E, Kesler A (2015). Evidence of multidomain mild cognitive impairment in idiopathic intracranial hypertension. Journal of neuro-ophthalmology: the official journal of the North American Neuro-Ophthalmology Society.

[52] Alperin N (2013). MRI evidence of impaired CSF homeostasis in obesity-associated idiopathic intracranial hypertension. AJNR. American journal of neuroradiology.

[53] Zatorre RJ, Fields RD, Johansen-Berg H (2012). Plasticity in gray and white: neuroimaging changes in brain structure during learning. Nature neuroscience.

[54] Peer M, Salomon R, Goldberg I, Blanke O, Arzy S (2015). Brain system for mental orientation in space, time, and person. Proceedings of the National Academy of Sciences of the United States of America.

[55] Young LR, Shelhamer M (1990). Microgravity enhances the relative contribution of visually-induced motion sensation. Aviation, space, and environmental medicine.

[56] Landi SM, Baguear F, Della-Maggiore V (2011). One week of motor adaptation induces structural changes in primary motor cortex that predict long-term memory one year later. The Journal of neuroscience: the official journal of the Society for Neuroscience.

[57] Seidler RD, Mulavara AP, Bloomberg JJ, Peters BT (2015). Individual predictors of sensorimotor adaptability. Frontiers in systems neuroscience.

[58] Ostry DJ, Gribble PL (2016). Sensory Plasticity in Human Motor Learning. Trends in neurosciences.

[59] Lee HK, Whitt JL (2015). Cross-modal synaptic plasticity in adult primary sensory cortices. Current opinion in neurobiology.

[60] Gill TM, Allore H, Guo Z (2004). The deleterious effects of bed rest among community-living older persons. *The journals of gerontology*. Series A, Biological sciences and medical sciences.

[61] Kortebein P (2008). Functional impact of 10 days of bed rest in healthy older adults. *The journals of gerontology*. Series A, Biological sciences and medical sciences.

[62] Albi A (2016). Free water elimination improves test-retest reproducibility of diffusion tensor imaging indices in the brain: A longitudinal multisite study of healthy elderly subjects. Human brain mapping.

