# Supplementary material for: Intracranial Fluid Redistribution But No White Matter Microstructural Changes During a Spaceflight Analog
Source: Sci Rep. 2017 Jun 9;7:3154. doi: 10.1038/s41598-017-03311-w (PMC5466616; doi:10.1038/s41598-017-03311-w)
Supplement: Supplementary file 1 — Supplementary Figure and Tables [file 41598_2017_3311_MOESM1_ESM.pdf]

Supplementary Material To:

Title: Intracranial Fluid Redistribution But No White Matter Microstructural Changes During a Spaceflight Analog

Authors: Vincent Koppelmans[1], Ofer Pasternak[2], Jacob J. Bloomberg[3], Yiri E. De Dios[4], Scott J. Wood[3,5], Roy Riascos[6], Patricia A. Reuter-Lorenz[7], Igor S. Kofman[4], Ajitkumar P. Mulavara[4], Rachael D. Seidler[1,7,8*]

Authors' affiliations: [1] School of Kinesiology, University of Michigan, Ann Arbor, MI, US; [2] Departments of Psychiatry and Radiology, Brigham and Women's Hospital and Harvard Medical School, Boston, MA, US; [3] NASA Johnson Space Center, Houston, TX, US; [4] KBRwyle, Houston, TX, US; [5] Azusa Pacific University, Azusa, CA, US; [6] The University of Texas Health Science Center, Houston, TX, US; [7] Department of Psychology, University of Michigan, Ann Arbor, MI, US; [8] Neuroscience Program, University of Michigan, Ann Arbor, MI, US

[*] Corresponding author

Neuromotor Behavior Laboratory, University of Michigan

401 Washtenaw Ave.

Ann Arbor, MI, 48109-2214, US

rseidler@umich.edu

Similarities between A) Free Water Distribution Changes from pre- to post-Bed Rest, B) Gray Matter Volume Changes from pre- to post-Bed Rest, and C) Gray Matter Volume Changes from pre- to post-Spaceflight

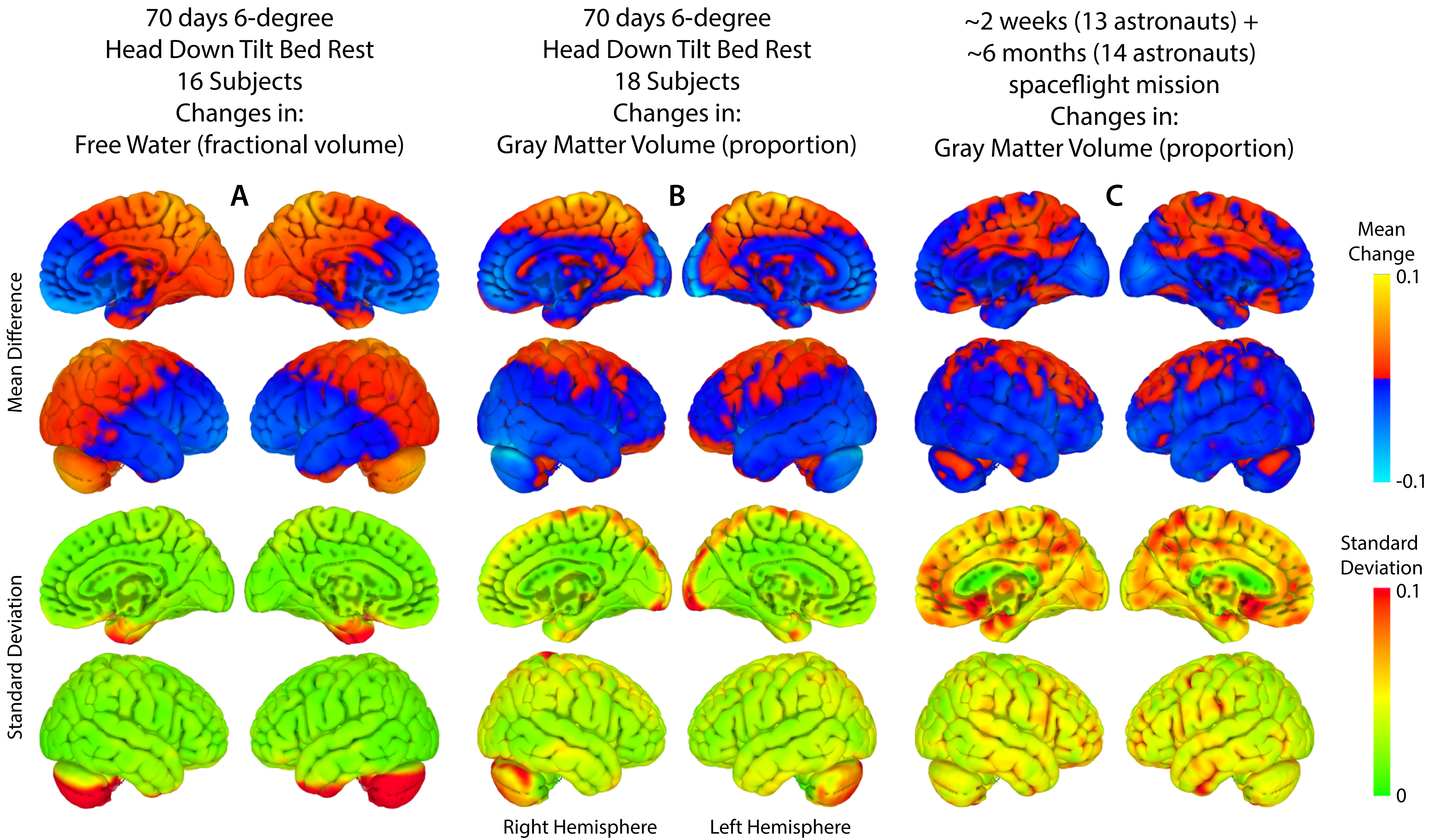

Supplementary Figure Captions

Supplementary Figure 1. Qualitative comparison of Free Water and Gray Matter changes over the course of HDBR and Gray Matter changes from pre- to post-spaceflight

Mean difference images (top 2 rows) show the average of the difference images (8 days pre-HDBR to ~70 days in HDBR, or pre-spaceflight to post-spaceflight). Standard deviation images (bottom 2 rows) show the standard deviation over the difference images of all subjects. Over groups, mean difference images and standard deviation images have been scaled identically for comparison. A (left two columns)= Free Water changes from 8 days pre-HDBR to ~70 days in HDBR (data from the current report); B (middle two columns)= Gray Matter changes from 8 days pre-HDBR to ~70 days in HDBR (Koppelmans et al., npj Microgravity 2016; doi:10.1038/s41526-016-0001-9; same participants as A); C (right two columns)= Gray Matter changes from pre- to post-Spaceflight (Koppelmans et al., npj Microgravity 2016; doi:10.1038/s41526-016-0001-9)

Supplementary tables to:

Intracranial Fluid Redistribution and White Matter Microstructural Changes During a Spaceflight Analog

Vincent Koppelmans, Ofer Pasternak, Jacob J. Bloomberg, Yiri E. De Dios, Scott J. Wood,
Patricia A. Reuter-Lorenz, Igor S. Kofman, Ajitkumar P. Mulavara, Rachael D. Seidler

This sheet contains an overview of locations in which we observed free water changes:

1) HBDR vs. Normative: Free Water Increase (non-parametric)
2) HDBR vs. Normative Free Water Decrease (non-parametric)
3) Δ Contrast: Free Water Increase with HDBR *
4) Δ Contrast: Free Water Decrease with HDBR *

Anatomical labels of all peak voxels with a minimum distance of 1cm within clusters showing significant (family wise error corrected) free water changes are reported. Coordinates refer to the MNI coordinate system in mm. Changes reflect changes in free water from pre-HDBR to the end of HDBR relative to changes in normative control subjects over a similar time interval.

* The Δ contrast is described in the manuscript (see Figure 1B). In terms of focal free water it assumes a stable baseline (no changes before bed rest) with increases/decreases during bed rest and recovery post bed rest.

Regions highlighted in green are obtained from the **Harvard-Oxford Cortical Structural Atlas**
Regions highlighted in green are obtained from the **Harvard-Oxford Subcortical Structural Atlas**
Regions highlighted in orange are obtained from the **Cerebellar Atlas in MNI152 (normalization with FNIRT)**
These atlasses are bundeld with Oxford Centre for Functional MRI of the Brain Software Library
http://fsl.fmrib.ox.ac.uk/fsl/fslwiki/FSL
http://fsl.fmrib.ox.ac.uk/fsl/fslwiki/Atlases

4) Δ Contrast: Free Water Decrease with HDBR *

Regions in which Free Water was stable at baseline, DECREASED from pre-HDBR to ~70 Days in HDBR and recovered post-HDBR (also see Figure 2B)

X	Y	Z	Brain Region
28	-72	44	Lateral Occipital Cortex; superior division
1	-51	55	Precuneous Cortex
0	-60	45	Precuneous Cortex
1	-41	59	Precuneous Cortex
37	-53	51	Angular Gyrus
12	-77	36	Cuneal Cortex
-32	-53	48	Superior Parietal Lobule
-26	-73	42	Lateral Occipital Cortex; superior division
40	-38	52	Superior Parietal Lobule
-11	-80	37	Cuneal Cortex
-23	-29	65	Postcentral Gyrus
-32	-27	60	Postcentral Gyrus
-43	-35	50	Postcentral Gyrus
29	-28	64	Postcentral Gyrus
14	-31	72	Postcentral Gyrus
-2	-76	32	Precuneous Cortex
-22	-42	64	Postcentral Gyrus
-30	-40	57	Superior Parietal Lobule
25	-40	63	Superior Parietal Lobule
0	-29	55	Precentral Gyrus
-25	-85	25	Lateral Occipital Cortex; superior division
24	-90	23	Occipital Pole
2	-19	67	Precentral Gyrus
1	0	65	Juxtapositional Lobule Cortex (formerly Supplementary Motor Cortex)
-12	-73	48	Lateral Occipital Cortex; superior division
-6	-95	17	Occipital Pole
-41	-14	49	Precentral Gyrus
2	-89	20	Occipital Pole
0	-12	47	Cingulate Gyrus; anterior division
29	-3	58	Middle Frontal Gyrus
0	3	49	Juxtapositional Lobule Cortex (formerly Supplementary Motor Cortex)
-8	-94	-2	Occipital Pole
46	-66	35	Lateral Occipital Cortex; superior division
10	-85	6	Intracalcarine Cortex
37	-87	2	Lateral Occipital Cortex; inferior division
-19	-95	11	Occipital Pole
-1	-70	-22	Vermis VI
21	-81	-25	Right Crus I
-10	-63	-8	Lingual Gyrus
37	-74	-27	Right Crus I
-31	-73	-24	Left Crus I
43	-13	44	Precentral Gyrus
-45	-63	27	Lateral Occipital Cortex; superior division
-26	-2	54	Middle Frontal Gyrus
-29	-89	5	Lateral Occipital Cortex; inferior division
-44	-70	18	Lateral Occipital Cortex; superior division

Counts	
1	Angular Gyrus
1	Cingulate Gyrus; anterior division
2	Cuneal Cortex
1	Intracalcarine Cortex
2	Juxtapositional Lobule Cortex
2	Lateral Occipital Cortex; inferior division
7	Lateral Occipital Cortex; superior division
1	Left Crus I
1	Lingual Gyrus
2	Middle Frontal Gyrus
5	Occipital Pole
6	Postcentral Gyrus
4	Precentral Gyrus
4	Precuneous Cortex
2	Right Crus I
4	Superior Parietal Lobule
1	Vermis VI

3) Δ Contrast: Free Water Increase with HDBR *

Regions in which Free Water was stable at baseline, INCREASED from pre-HDBR to ~70 Days in HDBR and recovered post-HDBR (also see Figure 2B)

X	Y	Z	Brain Region
0	42	-11	Paracingulate Gyrus
-33	20	-8	Insular Cortex
-38	24	0	Frontal Operculum Cortex
43	20	1	Frontal Operculum Cortex
29	55	2	Frontal Pole
38	45	-11	Frontal Pole
34	35	-12	Frontal Pole
-25	55	1	Frontal Pole
-28	38	-11	Frontal Pole
1	38	6	Cingulate Gyrus; anterior division
44	41	0	Frontal Pole
-50	6	-2	Central Opercular Cortex
-39	45	-5	Frontal Pole
-40	10	1	Insular Cortex
-39	43	8	Frontal Pole
33	10	-22	Temporal Pole
49	3	-2	Planum Polare
23	38	-17	Frontal Pole
12	23	3	Right Caudate
56	-1	6	Central Opercular Cortex
1	22	-20	Subcallosal Cortex
54	13	9	Inferior Frontal Gyrus; pars opercularis
40	15	-13	Insular Cortex
1	16	-8	Subcallosal Cortex
-55	-7	9	Central Opercular Cortex
-46	31	5	Inferior Frontal Gyrus; pars triangularis
-36	16	-26	Temporal Pole
-30	7	-22	Temporal Pole
17	4	-21	Parahippocampal Gyrus; anterior division
-12	21	-18	Subcallosal Cortex
-43	-3	9	Insular Cortex
53	4	-24	Middle Temporal Gyrus; anterior division
55	-5	-16	Middle Temporal Gyrus; anterior division
48	-2	-36	Inferior Temporal Gyrus; anterior division
62	-18	-4	Superior Temporal Gyrus; posterior division
-55	-12	-10	Superior Temporal Gyrus; posterior division

Counts	
3	Central Opercular Cortex
1	Cingulate Gyrus; anterior division
2	Frontal Operculum Cortex
9	Frontal Pole
1	Inferior Frontal Gyrus; pars opercularis
1	Inferior Frontal Gyrus; pars triangularis
1	Inferior Temporal Gyrus; anterior division
4	Insular Cortex
2	Middle Temporal Gyrus; anterior division
1	Paracingulate Gyrus
1	Parahippocampal Gyrus; anterior division
1	Planum Polare
1	Right Caudate
3	Subcallosal Cortex
2	Superior Temporal Gyrus; posterior division
3	Temporal Pole

2) HDBR vs. Normative Free Water Decrease (non-parametric)

Regions with Focal Free Water DECREASES from pre-HDBR to ~70 Days in HDBR relative to Free Water Changes in Normative Subjects over a similar time course (also see Figure 2A)

X	Y	Z	Brain Region
30	-75	44	Lateral Occipital Cortex; superior division
36	-51	53	Superior Parietal Lobule
-44	-50	48	Supramarginal Gyrus; posterior division
18	-60	59	Lateral Occipital Cortex; superior division
-44	-35	51	Postcentral Gyrus
42	-43	54	Superior Parietal Lobule
-6	-73	29	Precuneous Cortex
2	-46	46	Precuneous Cortex
9	-77	34	Cuneal Cortex
-10	-82	36	Cuneal Cortex
-25	-71	44	Lateral Occipital Cortex; superior division
-2	-37	44	Cingulate Gyrus; posterior division
-32	-26	60	Postcentral Gyrus
-33	-43	57	Superior Parietal Lobule
-19	-52	62	Superior Parietal Lobule
-19	-27	61	Precentral Gyrus
-23	-38	62	Postcentral Gyrus
31	-38	57	Postcentral Gyrus
49	-50	44	Angular Gyrus
-15	-31	71	Postcentral Gyrus
0	-62	43	Precuneous Cortex
14	-50	66	Superior Parietal Lobule
-17	-72	50	Lateral Occipital Cortex; superior division
-19	-61	57	Lateral Occipital Cortex; superior division
0	-86	31	Cuneal Cortex
-27	-84	24	Lateral Occipital Cortex; superior division
32	-28	62	Postcentral Gyrus
-43	-67	32	Lateral Occipital Cortex; superior division
4	-40	64	Postcentral Gyrus
-3	-94	11	Occipital Pole
32	-83	23	Lateral Occipital Cortex; superior division
-2	-60	61	Precuneous Cortex
1	-4	55	Juxtapositional Lobule Cortex (formerly Supplementary Motor Cortex)
-17	-88	26	Lateral Occipital Cortex; superior division
-10	-94	-1	Occipital Pole
21	-91	24	Occipital Pole
11	-85	4	Intracalcarine Cortex
2	-10	46	Cingulate Gyrus; anterior division
10	-96	13	Occipital Pole
-13	-84	2	Intracalcarine Cortex
-15	-67	23	Precuneous Cortex
-41	-14	47	Precentral Gyrus
20	-98	-1	Occipital Pole
-2	-20	71	Precentral Gyrus
2	-31	68	Precentral Gyrus
-21	-95	-3	Occipital Pole
23	-97	9	Occipital Pole
18	-31	60	Precentral Gyrus
55	-40	41	Supramarginal Gyrus; posterior division
-11	14	65	Superior Frontal Gyrus
16	-31	73	Postcentral Gyrus
-27	-90	5	Lateral Occipital Cortex; inferior division
55	-60	29	Lateral Occipital Cortex; superior division
-5	23	62	Superior Frontal Gyrus
-17	-96	8	Occipital Pole
54	-47	28	Angular Gyrus
-15	-10	70	Superior Frontal Gyrus
-52	-51	33	Angular Gyrus
43	-65	41	Lateral Occipital Cortex; superior division
8	8	68	Superior Frontal Gyrus
-46	-59	21	Angular Gyrus
-14	8	39	Paracingulate Gyrus
-36	-82	7	Lateral Occipital Cortex; inferior division
40	-81	8	Lateral Occipital Cortex; inferior division
-17	-77	-23	Left Crus I
-3	-65	-18	Vermis VI
5	-62	-10	Right V
28	-79	-31	Right Crus I
14	-83	-25	Right Crus I
-13	-62	-9	Lingual Gyrus
-12	-72	-15	Left VI
1	-73	-34	Vermis Crus II
-23	-69	-22	Left VI
-32	-75	-24	Left Crus I
18	-67	-13	Lingual Gyrus
-2	-78	-16	Vermis VI
38	-72	-16	Occipital Fusiform Gyrus
-24	-87	-17	Occipital Fusiform Gyrus
-47	-60	-22	Temporal Occipital Fusiform Cortex

Counts	
4	Angular Gyrus
1	Cingulate Gyrus; anterior division
1	Cingulate Gyrus; posterior division
3	Cuneal Cortex
2	Intracalcarine Cortex
1	Juxtapositional Lobule Cortex
3	Lateral Occipital Cortex; inferior division
11	Lateral Occipital Cortex; superior division
2	Left Crus I
2	Left VI
2	Lingual Gyrus
2	Occipital Fusiform Gyrus
8	Occipital Pole
1	Paracingulate Gyrus
8	Postcentral Gyrus
5	Precentral Gyrus
5	Precuneous Cortex
2	Right Crus I
1	Right V
4	Superior Frontal Gyrus
5	Superior Parietal Lobule
2	Supramarginal Gyrus; posterior division
1	Temporal Occipital Fusiform Cortex
1	Vermis Crus II
2	Vermis VI

1) HBDR vs. Normative: Free Water Increase (non-parametric)

Regions with Focal Free Water INCREASES from pre-HDBR to ~70 Days in HDBR relative to Free Water Changes in Normative Subjects over a similar time course (also see Figure 2A)

X	Y	Z	Brain Region
-43	32	-2	Frontal Orbital Cortex
-1	52	-13	Frontal Medial Cortex
42	43	-2	Frontal Pole
-47	21	4	Frontal Operculum Cortex
0	52	4	Paracingulate Gyrus
50	20	8	Inferior Frontal Gyrus; pars opercularis
39	21	-8	Frontal Orbital Cortex
0	40	-19	Frontal Medial Cortex
-27	40	-9	Frontal Pole
35	55	4	Frontal Pole
-37	46	7	Frontal Pole
-46	30	13	Inferior Frontal Gyrus; pars triangularis
8	49	14	Paracingulate Gyrus
-25	56	3	Frontal Pole
-35	19	-7	Insular Cortex
32	33	-11	Frontal Orbital Cortex
-35	49	-8	Frontal Pole
23	57	-5	Frontal Pole
42	42	9	Frontal Pole
53	7	-20	Temporal Pole
53	9	8	Precentral Gyrus
1	37	4	Cingulate Gyrus; anterior division
-29	54	12	Frontal Pole
66	-23	-13	Middle Temporal Gyrus; posterior division
49	31	15	Inferior Frontal Gyrus; pars triangularis
59	-5	-12	Middle Temporal Gyrus; anterior division
-40	10	1	Insular Cortex
50	6	-4	Planum Polare
53	-1	7	Central Opercular Cortex
14	58	-10	Frontal Pole
24	40	-14	Frontal Pole
55	-6	-31	Middle Temporal Gyrus; anterior division
-52	10	8	Inferior Frontal Gyrus; pars opercularis
-2	44	26	Paracingulate Gyrus
15	63	11	Frontal Pole
-54	-6	7	Central Opercular Cortex
63	-10	-4	Superior Temporal Gyrus; posterior division
-9	61	14	Frontal Pole
-52	8	-2	Temporal Pole
10	45	26	Paracingulate Gyrus
-30	46	27	Frontal Pole
19	60	22	Frontal Pole
14	64	1	Frontal Pole
24	2	-16	Right Amygdala
41	14	-27	Temporal Pole
30	42	30	Frontal Pole
-38	8	-16	Insular Cortex
59	2	15	Precentral Gyrus

Counts	
2	Central Opercular Cortex
1	Cingulate Gyrus; anterior division
2	Frontal Medial Cortex
1	Frontal Operculum Cortex
3	Frontal Orbital Cortex
17	Frontal Pole
2	Inferior Frontal Gyrus; pars opercularis
2	Inferior Frontal Gyrus; pars triangularis
3	Insular Cortex
2	Middle Temporal Gyrus; anterior division
1	Middle Temporal Gyrus; posterior division
4	Paracingulate Gyrus
1	Planum Polare
2	Precentral Gyrus
1	Right Amygdala
1	Superior Temporal Gyrus; posterior division
3	Temporal Pole
